# Utilizing video on myocardial infarction as a health educational intervention in patient waiting areas of the developing world: A study at the emergency department of a major tertiary care hospital in India

**DOI:** 10.1186/1755-7682-1-14

**Published:** 2008-07-29

**Authors:** Naveen Dhawan, Omar Saeed, Vineet Gupta, Rishi Desai, Melvin Ku, Sanjeev Bhoi, Sanjay Verma

**Affiliations:** 1 Department of Medicine, UCLA, Los Angeles, CA, USA; 2 Department of Medicine, Emory University, Atlanta, GA, USA; 3 Department of Emergency Medicine (JPNATC), All India Institute of Medical Sciences (AIIMS), New Delhi, India; 4 Department of Infectious Diseases, University of Southern California, Keck School of Medicine, Los Angeles, CA, USA; 5 UCLA School of Public Health, University of Southern California, Los Angeles, CA, USA; 6 Department of Medicine, Michigan State University, East Lansing, MI, USA

## Abstract

**Objective:**

To study the effect of health educational video instruction on increasing patients' knowledge in a hospital waiting area of a developing country.

**Methods:**

An educational video on signs, symptoms, and risk factors of myocardial infarction (MI) was played in an Emergency Department (ED) patient waiting area of an urban tertiary care hospital in India. Participants (n = 217) were randomly assigned to two groups: an intervention group that viewed the MI video (n = 111) and a control group that did not view the video (n = 106). Each group took a standard survey of thirty-seven questions to assess baseline knowledge pertaining to MI (pretest). The intervention group then viewed the video and the initial survey was re-administered to each group (posttest).

**Results:**

At baseline (pretest) there was no statistically significant difference between the intervention and control group in the mean number of correct (18.1 vs. 19.0, p = 0.19), incorrect (9.4 vs. 8.6, p = 0.27) and unsure (9.6 vs. 9.3, p = 0.78) responses per participant. After viewing the video on MI, the intervention group had a statistically significant improvement in the mean number of correct responses (27.0 vs. 20.0, p < 0.001), and a significant decline in the mean number of unsure responses (1.8 vs. 9.4, p < 0.001) compared to the posttest responses of the control group. There was no significant change in the number of incorrect responses on the posttest between the intervention and control groups, (8.3 vs. 7.7, p = 0.35), respectively.

**Conclusion:**

A health educational video can serve as an effective tool for increasing patients' short-term knowledge and awareness of health conditions in a hospital waiting area of a developing country.

**Practice Implications:**

Health educational videos serve as a public health low cost intervention that demonstrates clear short term benefits. Health care workers in developing countries can help educate individuals presenting to hospitals by displaying these videos in hospital waiting areas.

## 1. Introduction

As a developing country, India has witnessed unprecedented socioeconomic growth in the past few decades. However, the Indian healthcare system has struggled to keep up with the increasing demands in an era of rapid medical and technological advancement [1].

Healthcare delivery in India occurs in a variety of rural and urban settings, with a majority of patients who are poor, illiterate, and have limited access to health information [2]. Patients often turn to indigenous systems of medicine and untrained healthcare providers; even during serious medical events [3,4].

The lack of recognition of a serious medical event among populations in the developing world is well documented. One study in northwest India demonstrated that most patients lacked awareness of basic signs and symptoms of stroke [5]. Another study conducted among stroke patients and those accompanying them had similar findings and suggested that audiovisual interventions may help to remedy the problem [6]. In the developing world, video interventions are particularly helpful at teaching poorly educated, illiterate populations about health issues [7].

Several studies have been conducted to formally address the role of educational videos in disseminating information to patients. Video-based patient information provided prior to percutaneous cardiac interventions has shown effectiveness in improving patients' short term knowledge about coronary angiography and angioplasty [8]. Educational videos have shown success in a variety of clinical settings including Emergency Department (ED) waiting rooms [9-11] and outpatient clinics. In the urban U.S. hospital ED setting, Williams *et al*. [12] demonstrated an increase in knowledge among patients who were shown an educational video on heart disease. As far as implementing educational intervention in the developing world, Hubley [13] established that usage of video is an effective tool.

While many studies have demonstrated the utility of videos as educational tools in industrialized countries, further exploration is needed for developing nations, particularly in hospital patient waiting areas. The importance of establishing effective educational tools was underscored by a recent study in Pakistan that reported a lack of knowledge of modifiable risk factors of heart disease in patients who had experienced an acute myocardial infarction (MI) [14]. We studied the use of a health educational video on MI in a patient waiting area of the ED at an urban tertiary care academic hospital in India. The objective was to study the effect of health educational video instruction on increasing patients' knowledge and short-term retention in a hospital waiting area.

## 2. Materials and methods

### 2.1 Study Type

This is a prospective randomized intervention study designed to evaluate the effect of a video on short term knowledge pertaining to myocardial infarction.

### 2.2. Study site

Within India, the risk of coronary artery disease is greatest in urban settings [15], so we selected the Emergency room of a major tertiary care urban health center for this study. All India Institute of Medical Sciences (AIIMS) is a large government funded tertiary care academic hospital and research center in New Delhi, India with a daily census of about 600 patients in the ED. The busy environment limits physicians in spending adequate time educating patients about healthy lifestyle choices and prevention. It was thus determined that the use of health educational videos in a hospital such as AIIMS may be especially useful in supplementing patients' knowledge during their visits. Furthermore, ED participants were chosen in this study to sample people from a variety of backgrounds to give a closer representation of the population at large. A heterogeneous population enabled measurement of the video's favorable impact over people of broad backgrounds and shows that it is widely applicable.

Multiple ED waiting areas are scattered throughout the AIIMS hospital, and one of these was identified for conducting the study. This waiting area was a section in the ED where patients with lower acuity are seated.

### 2.3 Participants

Individuals were selected through a convenience sampling technique. Patients and their family members aged 18 years or above, able to read or understand Hindi, present in the waiting area were eligible for inclusion in the study. Of the 238 people approached by the study team over a seven day period, 217 people met inclusion criteria and gave the oral informed consent to proceed with the study.

### 2.4 Survey questionnaire

The Pretest survey instrument consisted of 37 knowledge-based non validated questions assessing the participant's level of understanding on various aspects of MI. It contained 7 stand alone questions assessing basic knowledge of MI and three separate tables asking respondents to mark symptoms, risk factors, and therapeutic options pertaining to MI (Appendix 1–2). Each question allowed the participant to respond as "yes," "no," or "unsure." Demographic questions addressed age, gender, educational level, languages read and spoken, type of family (nuclear or extended), past history of heart attack, family history of hypertension, frequency of exercise, and history of tobacco/smoking.

The posttest, given to both groups, was identical to the pretest with the exception of the demographic questions being omitted. Additionally, the posttest given to the intervention group which viewed the video, contained nine questions soliciting participants' attitudes and perceptions regarding the video, its content, feasibility, and the atmosphere in which it was shown (Appendix 3).

This questionnaire was developed using the direct English transcription from the video based on the theory of Bloom [16] who has purported that the question *cues *appropriate for knowledge-base questions include lists and identifying variables and that mastery of a subject can be obtained through the observation and recall of information. Knowledge was thus assessed through various questions in both the pre-intervention questionnaire and the post-intervention questionnaire.

The questionnaire was originally composed in English and later translated to the Hindi language (the national language of India). Translations in Hindi were checked by bilingual speakers to assure that questions intentionally worded as "negatives" remained appropriately meaningful. Furthermore, individuals who only spoke Hindi verified correct impression and idiomatic meaning of each question to the research team prior to survey administration.

### 2.5 The Interventional Video

A video pertaining to the topic of MI was chosen because the wide prevalence of cardiovascular disease among the Indian population has been well-established [17,18]. In addition, MI is associated with minimal stigma and cultural overlays, general familiarity in the population, and applicability to adults of both genders, allowing for more homogeneity among participants. Moreover, increasing general knowledge of MI is an important goal, because timely recognition of symptoms and subsequent interventions has been shown to decrease morbidity and mortality [19-21].

The video was first produced for the study based on the format of existing health videos in English [22]. The principles were in accordance with guidelines from the American Heart Association and the Cardiology Society of India. A U.S. based practicing Indian cardiologist was videotaped providing information in Hindi, about the signs, symptoms, treatment, risk factors and prevention of an MI. A heart model was used for describing basic heart physiology and the video included a standardized patient encounter involving an actor (of Indian origin) discussing risk factors, signs, symptoms and treatment options correlating to the diagnosis of MI. The video was five minutes in length.

### 2.6 Survey administration procedure

Consenting individuals were randomized to either the control group or the intervention group. Randomization was done using computer generated sequences, assigning participants to either of the two groups. Both groups were administered identical pretest questionnaires. Then, the intervention group was taken to a separate room and shown the interventional video. After the 5-minute video ended, both control and intervention groups were given posttest questionnaires. The research team emphasized that discussion of responses should not occur between subjects and this was evident by no observed discussion amongst participants during survey completion.

**Figure 1:**
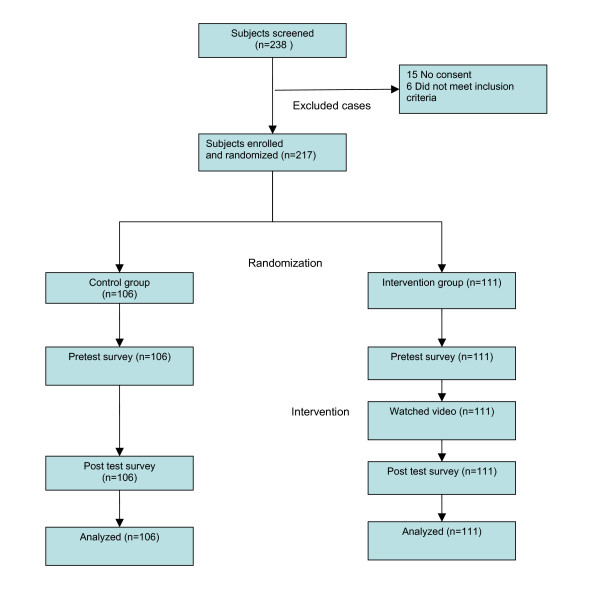
**Flow diagram depicting the study design.** Baseline demographic characteristics of participants in control and intervention groups.

Data collection for this study occurred over a period of seven consecutive days with multiple survey administration and video intervention sessions during each day. For each session, the sequence of participant selection, randomization, group segregation, and survey administration was identical. Overall, there were 106 controls and 111 subjects in the intervention group, for a total 217 participants (see Figure 1). After all members of both groups completed posttests, these surveys were collected and correct answers were explained.

To ensure uniformity in questionnaire administration among both literate and illiterate subjects, a member of the research team read a standard script of the pretest and posttest aloud for illiterate participants.

All data from pretests and posttests were compiled and analyzed by members of the research team that were unaware if a particular survey was done by a participant in the control or intervention group. Institutional review board approval was obtained from AIIMS prior to initiation of this study.

### 2.7 Statistical analysis

Statistically significant differences in demographics of both intervention and control groups were calculated using chi squares test for categorical variables and 2-sample t-tests for continuous variables. 2 sample t-tests was also used to determine statistically significant differences in the mean number of correct, incorrect and unsure responses per participant between pretest and posttest for both groups. All statistical calculations were done by Graph Pad In Stat 3.0 software. Differences were considered statistically significant at p < 0.05.

### 3. Results

Analysis of demographic data showed no significant differences between control and intervention groups on all measured variables, except "years of formal education" (with the intervention group having a higher level of education) and "history of hypertension" (with a greater number of participants in the intervention group reporting that they lacked knowledge of whether or not they had hypertension). Analyses shown are done after adjustment for education levels and hypertension history (See Table 1).

**Table 1:** Baseline demographic characteristics of participants in control and intervention groups.

Characteristic	Control group n = 106	Intervention group n = 111	P value
Age	35.6 (SD 10.6)	34.8 (SD 10.3)	0.75
Gender			0.1
-Male	84 (79)	93 (84)	
-Female	22 (21)	18 (16)	
Role in emergency department:			0.75
-Patient	35 (33)	35 (32)	
-Family member	56 (53)	58 (52)	
-Friend/caretaker	15 (14)	18 (16)	
Mother tongue			0.42
-Hindi	105 (99)	109 (98)	
-Other	1 (1)	2 (2)	
Family characteristics			0.55
-Nuclear family	20 (19)	22 (20)	
-Extended family	85 (80)	89 (80)	
Years of formal education			**0.024**
-None			
-< 5	0 (0)	1 (1)	
-6–10	2 (2)	7 (6)	
-11–12	12 (11)	15 (14)	
-College	51 (48)	40 (36)	
-Graduate school	25 (24)	36 (32)	
	16 (15)	12 (11)	
Hx of smoking/tobacco use?			0.25
-Yes	58 (55)	55 (50)	
-No	48 (45)	56 (50)	
Exercise			0.43
-None	53 (50)	52 (47)	
-Once a week	11 (10)	9 (8)	
-2–3 times a week	16 (15)	18 (16)	
-4–5 days a weeks	7 (7)	5 (5)	
-Daily	19 (18)	27 (24)	
Diabetes Mellitus			0.23
-Yes	0 (0)	2 (2)	
-No	84 (79)	82 (74)	
-Do not know	22 (21)	27 (24)	
Hypertension			**0.038**
-Yes	12 (11)	8 (7)	
-No	82 (78)	81 (73)	
-Do not know	12 (11)	22 (20)	
Family history of Hypertension?			0.25
-Yes	44 (42)	38 (34)	
-No	54 (51)	62 (56)	
-Do not know	8 (8)	11 (10)	
History of myocardial infarction?			0.076
-Yes	9 (9)	6 (5)	
-No	95 (89)	98 (88)	
-Do not know	2 (2)	7 (7)	

**2a** Comparison of the mean number of Correct responses amongst both groups. **2b** Comparison of the mean number of Incorrect responses amongst both groups. **2c** Comparison of the mean number of "unsure responses" amongst both groups.

At baseline (pretest), the mean numbers of correct, incorrect and unsure responses per participant for intervention versus control groups were as follows: (correct: 18.1 vs. 19.0, p = 0.19, incorrect: 9.4 vs. 8.6, p > 0.27, unsure: 9.6 vs. 9.3, p = 0.78), respectively. There was no statistically significant difference noted amongst the baseline knowledge pertaining to MI, with respect to our questionnaire between intervention and control groups.

**Figure 2:**
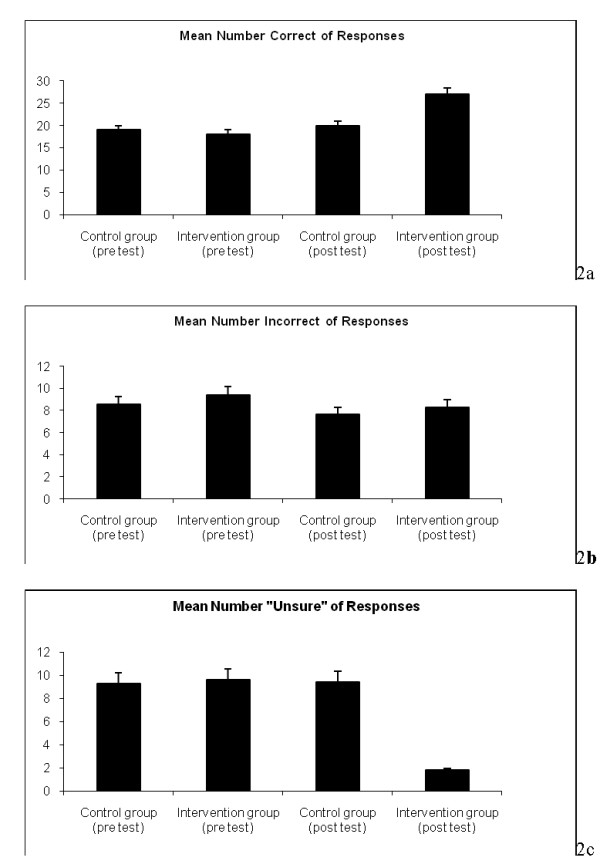
**Comparison of responses amongst both groups. 2a** shows mean number of correct responses per participant, along with standard error bars, for both control and intervention groups in their pretest and posttest.  No significant difference is observed between the pretest in the mean number of correct responses for control vs. intervention groups.  However, a statistically significant rise in mean number of correct responses is present in the posttest survey of the intervention group versus control, p< 0.001. **2b** shows mean number of incorrect responses per participant, along with standard error bars, for both control and intervention groups in their pretest and posttest.  No significant difference is observed in the mean number of incorrect responses for control vs. intervention groups. **2c** shows the mean number of unsure responses per participant, along with standard error bars, for both control and intervention groups in their pretest and posttest.  No significant difference is observed between the pretest mean of unsure responses for control vs. intervention groups.  However, a statistically significant decline in the mean number of correct responses is present in the posttest survey of the intervention group versus control, p< 0.001.

Posttest results showed significant changes in responses between intervention and control groups. The mean numbers of correct, incorrect, and unsure responses per participant for intervention versus control groups on posttest were as follows: (correct: 27.0 vs. 20, p < 0.001, incorrect: 8.3 vs. 7.7 p = 0.35, unsure: 1.8 versus 9.4, p < 0.001), respectively (See Table 2). There was a statistically significant increase in the number of correct responses and decline in the number of unsure responses for participants in the intervention groups in comparison to those who did not see the video (See Figure 2). No significant change was noted in the number of incorrect responses amongst posttest intervention and control groups (8.3 vs. 7.7, p = 0.35). Responses to the 9 questions that assessed the attitudes of viewers in terms of the video content and overall applicability are shown in Table 3.

**Table 2:** Numerical results comparing the mean number of correct, incorrect and "unsure" responses per participant

	Control	Intervention group	t test	Control	Intervention group	t test
	group (pre test) n = 106	(pre test) n = 111	p value control vs. intervention (pre test)	group (post test) n = 106	(post test) n = 111	p value control vs. intervention (post test)

Mean number of correct responses per participant	19.0 (95% CI: 17.8–20.1) SD: 5.4	18.1 (95% CI: 16.9–19.2) SD: 5.8	P = 0.19	20.0 (95% CI: 18.9–21.0) SD: 5.2	27.0 (95% CI: 25.9–28.1) SD: 5.5	P < 0.001
Mean number of incorrect responses per participant	8.6 (95% CI: 7.5–9.7) SD: 5.0	9.4 (95% CI: 8.2–10.5) SD: 4.8	P = 0.27	7.7 (95% CI: 6.6–8.7) SD: 4.6	8.3 (95% CI: 7.4–9.4) SD: 4.6	P = 0.35
Mean number of "unsure" responses per participant	9.3 (95% CI: 8.2–10.4) SD: 6.7	9.6 (95% CI: 8.5–10.7) SD: 7.3	P = 0.78	9.4 (95% CI: 8.3–10.5) SD: 6.7	1.8 (95% CI: 1.2–2.3)" SD: 3.0	P < 0.001

of both control and intervention groups. 95% confidence intervals are also shown, along with standard deviations (SD). Data are from pretest and posttest surveys given to each group. P-values are generated using the 2- samplet-test, and considered statistically significant at p < 0.05.

**Table 3:** Responses to questions assessing the participant's perspective after viewing the educational video on MI.

QUESTION 1: Did you find the information on the video helpful?	
Somewhat- 3	7 (6.3%)
Quite a deal- 4	51 (45.9%)
A great deal- 5	53 (47.7%)
QUESTION 2: Did you learn something you did not know about heart disease after watching the video?	
A little- 2	4 (3.6%)
Somewhat- 3	7 (6.3%)
Quite a bit-4	25 (22.5%)
A great deal-5	75 (67.6%)

QUESTION 3: Was the atmosphere in the emergency department a good place to view the video?	
Yes-1	99 (89.2%)
No-2	10 (9.0%)
Unsure-3	2 (1.8%)

QUESTION 4: Did you feel comfortable watching the video in the emergency department waiting room?	
Not at all- 1	9 (8.1%)
A little-2	4 (3.6%)
Somewhat-3	14 (12.6%)
Quite a bit-4	46 (41.4%)
A great deal- 5	38 (34.2%)

QUESTION 5: How would you rate the length of the video?	
Too short- 1	2 (1.8%)
A little short-2	8 (7.21%)
About right-3	82 (73.9%)
A little bit long-4	5 (4.5%)
Too long- 5	14 (12.6%)

QUESTION 6: Would you watch this video again if you could?	
Yes-1	109 (98.2%)
No-2	0 (0%)
Unsure-3	2 (1.8%)

QUESTION 7: Do you think you would get more information if this video was shown in a place other than the Emergency Department?	
Yes-1	31 (27.9%)
No-2	67 (60.4%)
Unsure-3	13 (11.7%)

QUESTION 8: After watching the video, do you think you will change any part of your current lifestyle to decrease your chances of heart disease?	
Not at all- 1	2 (1.8%)
A little-2	5 (4.5%)
Somewhat-3	20 (18.0%)
Quite a bit-4	47 (42.3%)
A great deal- 5	37 (33.3%)

QUESTION 9: Was it difficult to watch the video in the Emergency Department waiting room?	
Not at all- 1	72 (64.9%)
A little-2	26 (23.4%)
Somewhat-3	9 (8.1%)
Quite a bit-4	2 (1.8%)
A great deal- 5	2 (1.8%)

Results shown are percentages of the total numbers of participants choosing that response. Responses are from intervention group only (n = 111).

## 4. Discussion and conclusion

### 4.1. Discussion

Dissemination of information to patients in the developing world has been an ongoing public health struggle due to limited health care access and illiteracy. Our study aimed at using a unique form of technology to disseminate information and to study the effects of video on short-term educational benefits. Pandey *et al*. [23] recently demonstrated an increased utilization of health services following an intervention that informed poor populations of entitled health services. That study found that individuals were not aware of the resources they could utilize and called for educational interventions for improving the utilization of such resources. A video intervention may be particularly useful in rural non-hospital settings to inform individuals about the services provided at the hospital.

We hypothesized that viewing of health educational videos in patient waiting areas in developing countries is effective in the short-term. Our results show that waiting areas are effective places for visual health educational instruction through video.

There was a significant increase in the number of correct answers and a decrease in the number of unsure answers by participants in the intervention group after viewing the video. This strongly suggests that the video was useful in increasing the short term knowledge of signs and symptoms, prevention, and treatment of MI.

The average wait time at the ED of a major hospital similar to AIIMS in another developing country was found to be an average of 40 minutes prior to triage [24]. The instructional video utilizes a patient's wait for educational purposes. In our study, two third of participants felt that they learned something they did not know about heart disease after watching the video.

Many physicians are unable to spend time during the patient encounter to convey basic health concepts and preventive knowledge. Health instructional videos offer timely information so that patients can gain the most out of a physician visit by asking informed questions and having a basis upon which to understand the answers. In our study, all of the responders felt that they learned something valuable from the video, 98% believed that it would potentially lead to some form of behavioral change and one third of participants remarked that they would change their lifestyle "a great deal" after viewing the video. These high and idealistic percentages of positive feedback do raise a question of the veracity of participant's responses. It is unlikely that such a high percentage of participants will drastically alter their lifestyle based on a very short educational video. Perhaps a post intervention survey can be given to investigate how many people did alter their lifestyles. As far as the participant acceptability of this video, approximately ninety percent of the responders felt the ED setting was an effective venue for playing videos, and seventy five percent felt that the length of the video (5 minutes) was ideal. With ongoing research and feedback from viewers, the educational videos can be readily adapted to meet the changing needs of a medical center and the individuals that it serves.

Our study implicates that the placement of television sets and VCD players in clinical settings is beneficial. The expense for the entire setup in minimal and it requires minimal infrastructure to maintain over time.

This opens the possibility of creating health educational videos that are culture and disease specific, tailored to meet the needs of each particular region for each unique context in developing countries. Topics as wide-ranging as proper diet, exercise, motional/mental health, and STD prevention and treatment are all amenable to this form of patient education.

Our study has several notable limitations. First, this was a cross-sectional study and we did not administer follow-up questionnaires to determine whether there was long-term retention of information or change in health behaviors following the viewing of the video. Another delayed posttest after a month would be helpful in gauging the increase in long-term knowledge base of the participants. Second, administering identical knowledge based questions in the pretest and posttest may have skewed results because participants who watched the video may have paid particular attention to parts of the video that they knew were being tested.

Third, it is important to note that an experimental survey was used for our study. Such a survey enabled us to measure the *change *in knowledge related to several aspects of myocardial infarction after viewing an educational video. However, it is likely not a very useful tool in identifying specific knowledge strengths and weakness in the studied population. This is because specific domains are not defined and validated within the experimental survey.

Lastly, our study was conducted in a busy teaching hospital in New Delhi that serves as a tertiary care center for an economically impoverished patient base, and thus results may not be easily extrapolated to other populations. Further studies are needed to explore the utility of this health education tool in waiting areas of other hospital settings such as primary care or health centers in rural areas of the developing world.

### 4.2. Conclusion

This study suggests that health educational videos may have an applicable role in educating patients in hospital waiting areas of the developing world. Health educational videos when played in patient waiting areas of a developing country such as India have efficacy in short-term education of patients about health and medicine. In particular, the videos may help patients recognize key signs and symptoms of a critical condition. Implementation of brief educational health videos could provide patients information while they wait for their appointment, and in turn may improve the quality of physician visits.

A previous study conducted in the Emergency Department setting of a U.S. hospital demonstrated positive utility of a video was shown to patients to improve knowledge of coronary artery disease [11]. Our study is unique in that it reports such effectiveness of a video in the ED waiting area of a developing country. This study serves as an initial exploration of the utility of health educational videos in patient waiting areas of a developing country. Further studies are needed to elucidate the role of video in other health care settings and long-term retention of knowledge and behavioral change. If videos are found to be effective teaching instruments for these populations, culturally sensitive videos may be generated to meet the ever-changing needs of patient populations around the world. Future research can elucidate the challenges and barriers that may be present in such areas of hospitals. Furthermore, exploration is needed in determining variations in cultures that warrant an adaptation of such educational tools for respective regions throughout such countries.

### 4.3. Practice implications

This study aims to better utilize the time that patients spend in waiting areas. Since visual media is a tool with demonstrated efficacy for health education; videos played on televisions in these waiting areas have a high potential for meeting health educational objectives in a variety of clinical settings.

## Competing interests

ND heads an organization, Healing and Prevention Through Video (HPTV), that plans to produce health educational videos. The authors have no competing financial interests to disclose.

## Authors' contributions

ND and VG initially conceived the concept to test the impact of an educational video within a hospital setting in India. ND was involved with all aspects of this study. OS conducted data compilation, statistical analysis and revised initial drafts of the manuscript. VG, RD and MK were involved in writing and revising the manuscript. VG, SB, SV were part of the AIIMS research team in India that conducted the entire survey and assisted with completion of this investigation. All authors approved the final manuscript. The authors are part of the Developing World Health Promotion (DWHP) Study Group, a group of core researchers headed by ND that investigates health knowledge among global populations and studies the implementation of health promotional interventions in developing countries around the world.

## Appendix

See Tables 4, 5 and 6

**Table 4:** **Appendix 1.** Following are questions 1–8 from administered survey.

**1**.	**People who smoke cigarettes have a GREATER chance of having a heart attack**.
	_____True
	_____False
	_____I am not sure
**2**.	**People who exercise DECREASE their chances of having a heart attack**.
	_____True
	_____False
	_____I am not sure
**3**.	**Smoking DOES NOT affect your chances of having a heart attack**.
	_____True
	_____False
	_____I am not sure
**4**.	**ALL people have the SAME symptoms of a heart attack**.
	_____True
	_____False
	_____I am not sure
**5**.	**Chewing an aspirin as soon as you have signs of a heart attack is a GOOD thing**.
	_____True
	_____False
	_____I am not sure
**6**.	**When you have a heart attack, it DOES NOT matter WHEN you go to the hospital**.
	_____True
	_____False
	_____I am not sure
**7**.	**You can control your chances of having a heart attack by eating well**.
	_____True
	_____False
	_____I am not sure

**Table 5:** **Appendix 2.** Following are questions 11–37 from administered survey.

Which of the following are common symptoms of a heart attack?				
***Question Number***		***Yes***	***No***	***Unsure***

**8**	**Sudden pain or heaviness in chest**			
**9**	**Being drowsy**			
**10**	**Passing urine**			
**11**	**Pain going from chest to left arm or under the throat**			
**12**	**Blindness**			
**13**	**Sweating throughout the body**			
**14**	**Dizziness**			
**15**	**Increased hunger**			
**16**	**Difficulty in breathing**			
**17**	**Fever**			

Which of the following increases the risk of having a heart attack?				

***Question Number***		***Yes***	***No***	***Unsure***

**18**	**Smoking cigarettes**			
**19**	**Weight loss**			
**20**	**Obesity**			
**21**	**Depression**			
**22**	**Hypertension**			
**23**	**High cholesterol level in the blood**			
**24**	**Daily exercise**			
**25**	**Eating chocolates**			
**26**	**Sleeping too much**			
**27**	**Diabetes**			

Which of the following helps a person when they are having a heart attack?				

***Question Number***		***Yes***	***No***	***Unsure***

**28**	**Chewing aspirin**			
**29**	**Taking a short walk**			
**30**	**Eating fruits**			
**31**	**Drinking water**			
**32**	**Placing ice on chest**			
**33**	**Going immediately to the nearest hospital**			
**34**	**Sitting alone in a dark room**			
**35**	**Eating vegetables**			
**36**	**Receiving medication through a vein**			
**37**	**Drinking tea**			

**Table 6:** **Appendix 3.** Questions to access how the participant's perspective on viewing our educational video on MI in the Emergency department. Only given to the intervention group (n = 111).

**1. Did you find the information on the video helpful?**
______ Not at all
______ A little
______ Somewhat
______ Quite a bit
______ A great deal
**2. Did you learn something you did not know about heart disease after watching the video?**
______ Not at all
______ A little
______ Somewhat
______ Quite a bit
______ A great deal
**3. Was the atmosphere in the emergency department a good place to view the video?**
______ Yes
______ No
______ Unsure
**4. Did you feel comfortable watching the video in the emergency department waiting room?**
______ Not at all
______ A little
______ Somewhat
______ Quite a bit
______ A great deal
**5. How would you rate the length of the video?**
______ Too short
______ A little short
______ About right
______ A little bit long
______ Too long
**6. Would you watch this video again if you could?**
______ Yes
______ No
______ Unsure
**7. Do you think you would get more information if this video was shown in a place other than the Emergency Department?**
______ Yes
______ No
______ Unsure
**8. After watching the video, do you think you will change any part of your current lifestyle to decrease your chances of heart disease?**
______ Not at all
______ A little
______ Somewhat
______ Quite a bit
______ A great deal
**9. Was it difficult to watch the video in the Emergency Department waiting room?**
______ Not at all
______ A little
______ Somewhat
______ Quite a bit
______ A great deal

## References

[B1] Ramani KV, Mavalankar D (2006). Health system in India: opportunities and challenges for improvements. J Health Organ Manag.

[B2] Deogaonkar M (2004). Socio-economic inequality and its effect on healthcare delivery in India: inequality and healthcare. Electronic Journal of Sociology.

[B3] Singh P, Yadav RJ, Pandey A (2005). Utilization of indigenous systems of medicine & homoeopathy in India. Indian J Med Res.

[B4] Kumar R, Jaiswal V, Tripathi S, Kumar A, Idris MZ (2007). Inequity in health care delivery in India: the problem of rural medical practitioners. Health Care Anal.

[B5] Pandian JD, Jaison A, Deepak SS, Kalra G, Shamsher S, Lincoln DJ, Abraham G (2005). Public awareness of warning symptoms, risk factors, and treatment of stroke in northwest India. Stroke.

[B6] Das K, Mondal GP, Dutta AK, Mukherjee B, Mukherjee BB (2007). Awareness of warning symptoms and risk factors of stroke in the general population and in survivors stroke. J Clin Neurosci.

[B7] Bhave G, Lindan CP, Hudes ES, Desai S, Wagle U, Tripathi SP, Mandel JS (1995). Impact of an intervention on HIV, sexually transmitted diseases, and condom use among sex workers in Bombay, India. Aids.

[B8] Steffenino G, Viada E, Marengo B, Canale R (2007). Effectiveness of video-based patient information before percutaneous cardiac interventions. J Cardiovasc Med (Hagerstown).

[B9] Calderon Y, Haughey M, Bijur PE, Leider J, Moreno-Walton L, Torres S, Gennis P, Bauman LJ (2006). An educational HIV pretest counseling video program for off-hours testing in the emergency department. Ann Emerg Med.

[B10] Oermann MH, Webb SA, Ashare JA (2003). Outcomes of videotape instruction in clinic waiting area. Orthop Nurs.

[B11] Wei HG, Camargo CA (2000). Patient education in the emergency department. Acad Emerg Med.

[B12] Williams A, Lindsell C, Rue L, Blomkalns A (2007). Emergency Department education improves patient knowledge of coronary artery disease risk factors but not the accuracy of their own risk perception. Prev Med.

[B13] Hubley J (2006). Patient education in the developing world–a discipline comes of age. Patient Education and Counseling.

[B14] Khan MS, Jafary FH, Jafar TH, Fauqui AM, Rasool SI, Hatcher J, Chaturvedi N (2006). Knowledge of modifiable risk factors of heart disease among patients with acute myocardial infarction in Karachi, Pakistan: a cross sectional study. BMC Cardiovasc Disord.

[B15] Singh RB, Sharma JP, Rastogi V, Raghuvanshi RS, Moshiri M, Verma SP, Janus ED (1997). Prevalence of coronary artery disease and coronary risk factors in rural and urban populations of north India. EurHeart J.

[B16] Bloom B (1984). Taxonomy of educational objectives.

[B17] Goyal A, Yusuf S (2006). The burden of cardiovascular disease in the Indian subcontinent. Indian J Med Res.

[B18] Prabhakaran D, Shah P, Chaturvedi V, Ramakrishnan L, Manhapra A, Reddy KS (2005). Cardiovascular risk factor prevalence among men in a large industry of northern India. Natl Med J India.

[B19] Moser DK, Kimble LP, Alberts MJ, Alonzo A, Croft JB, Draqcup K, Evenson KR, Go AS, Hand MM, Kothari RU, Mensah GA, Morris DL, Pancioli AM, Riegel B, Zerwic JJ (2006). Reducing delay in seeking treatment by patients with acute coronary syndrome and stroke: a scientific statement from the American Heart Association Council on cardiovascular nursing and stroke council. Circulation.

[B20] The Thrombolysis in Myocardial Infarction (TIMI) trial (1985). Phase I findings. TIMI Study Group. N Engl J Med.

[B21] (1988). Randomised trial of intravenous streptokinase, oral aspirin, both, or neither among 17,187 cases of suspected acute myocardial infarction: ISIS-2. ISIS-2 (Second International Study of Infarct Survival) Collaborative Group. Lancet.

[B22] Gilder RS (1989). Fundamentals of video production and script writing. J Audiov Media Med.

[B23] Pandey P, Sehgal AR, Riboud M, Levine D, Goyal M (2007). Informing resource-poor populations and the delivery of entitled health and social services in rural India: a cluster randomized controlled trial. Jama.

[B24] Banerjea K, Carter AO (2006). Waiting and interaction times for patients in a developing country accident and emergency department. Emerg Med J.

